# Age at school entry and reported symptoms of attention-deficit/hyperactivity in first graders: results of the prospective cohort study ikidS

**DOI:** 10.1007/s00787-021-01813-7

**Published:** 2021-06-05

**Authors:** Christiane Diefenbach, Martina F. Schmidt, Michael Huss, Jochem König, Michael S. Urschitz, The ikidS Study Group, Dietmar Hoffmann, Maria Blettner, Annette Queisser-Wahrendorf, Awi Wiesel, Fred Zepp, Jörg Faber, Stephan Gehring, Eva Mildenberger, Stephan Letzel, Heike Elflein, Alexander K. Schuster, Brita Willershausen, Jens Weusmann, Christoph Matthias, Anne Läßig, Margarete Imhof, Perikles Simon

**Affiliations:** 1 Division of Paediatric Epidemiology, Institute of Medical Biostatistics, Epidemiology, and Informatics, University Medical Centre of the Johannes Gutenberg-University Mainz, Obere Zahlbacher Str. 69, 55131 Mainz, Germany; 2 Department of Child and Adolescence Psychiatry, University Medical Centre of the Johannes Gutenberg-University Mainz, Mainz, Germany

**Keywords:** Age at school entry, Attention-deficit/hyperactivity disorder, Oversupply, School

## Abstract

Young age at school entry (ASE) is related to attention-deficit/hyperactivity disorder in higher grades. The reason for this association is unclear, but medical oversupply and stress-related factors are discussed. We aimed to investigate whether ASE is associated with reported symptoms of attention-deficit/hyperactivity (ADH) already in first grade. Data of a population-based prospective cohort study (*N* = 2003; Mainz-Bingen region; Rhineland-Palatinate; Germany) with baseline assessments prior to school entry and two follow-ups during first grade were analysed. ADH symptoms were assessed by parent and teacher versions of the Strengths and Difficulties Questionnaire. Associations between ASE and scores of the hyperactivity/inattention subscale (range 0–10) were investigated by regression analysis and adjusted for potential confounders and baseline symptoms prior to school entry. In total, 1633 children (52% boys, mean ASE 6.5 years) were included. There were no relationships between ASE and parent-reported scores of the hyperactivity/inattention subscale prior to school entry and 3 months thereafter. However, at the end of first grade, ASE was negatively associated with the hyperactivity/inattention subscale in parent (− 0.7 subscale points per year ASE, standard error = 0.16, *p* < 0.0001) and teacher reports (− 1.2 subscale points per year ASE, standard error = 0.25, *p* < 0.0001). This ASE effect appeared more pronounced in girls than in boys. Young ASE is related to more reported symptoms of ADH at the end of first grade, but not before. The evolvement of this effect during first grade may be a clue to ASE-related stress factors.

**Supplementary Information:**

The online version contains supplementary material available at 10.1007/s00787-021-01813-7.

## Introduction

Numerous international studies have investigated the relationship between young age at school entry (ASE) and attention-deficit/hyperactivity disorder (ADHD). Two systematic reviews showed that the majority of studies provided evidence for an increased risk of the youngest children within a school class to receive an ADHD diagnosis and/or ADHD medication compared to their older classmates [1, 2]. In line with this observation, previous German studies have also found associations between relative ASE and rates of ADHD diagnoses and medical treatment [3] as well as ADHD-related symptoms [4–6]. In one study, ASE was associated with teacher-reported ADHD-related symptoms in the 2nd and 4th grades. The association remained after adjusting for potential confounders and prevalent symptoms at school entry and was stronger in the 2nd grade compared to the 4th grade [6].

In the literature, two major hypotheses for this relative age effect are discussed. The most common explanation considers the effect as caused by misinterpretation of conspicuous behaviour and unnecessary medical services in relatively younger children (i.e., oversupply hypothesis, e.g., [3, 7]). Because the youngest children within a grade are often developmentally less mature, they are more likely to behave more inattentively, more impulsively, and more hyperactively than their older classmates. Sustained attention and impulse control or motor inhibition are executive functions, which involve prefrontal structures. They are subject to maturational processes that occur particularly between preschool age and middle childhood [8, 9] and are found to be delayed in children with ADHD [9, 10]. According to this hypothesis, teachers presumably compare the younger children to others within the same grade rather than to children of the same age and, consequently, mistake their less developed executive functions—which are, in fact, age-appropriate—for ADHD symptoms. As a result, teachers are more likely to suggest a further diagnostic work-up of ADHD in younger children causing them to have a higher ADHD prevalence. In a US study, healthcare professionals reported that in almost half of the cases, teachers were the first to suggest the diagnosis of ADHD in a child [11].

An alternative explanation for the relative age effect assumes a causal biological stress mechanism (i.e., stress-related hypothesis, e.g., [6, 12]). As an important life event, school entry is associated with high cognitive, social, and emotional demands, requiring quick and appropriate adaptions to these demands within a few weeks after school entry. Some of the relatively younger and therefore developmentally more immature children may be disproportionately challenged by these demands, which could lead to increased stress, peer difficulties, and academic failure, which in turn could trigger or amplify ADHD or ADHD-like symptoms.

In this context, gender differences concerning the association between ASE and ADHD should be considered. It is well known that the prevalence of ADHD is higher in boys than in girls with a ratio of around 3:1 in population-based samples [13]. In clinical samples, even male-to-female ratios of up to 16:1 have been reported [14]. Among other factors, the developmental differences between classmates—which are central to both hypotheses on the relative age effect—might also affect the risk of receiving an ADHD diagnosis differently across gender. The oversupply hypothesis could apply to boys to a greater extent than to girls: boys are disadvantaged compared to girls concerning general maturation [15] and school readiness [16]. Thus, the youngest boys within a grade are the most developmentally immature pupils. Boys have a higher level of motor activity compared to girls [17], which decreases with age in both genders [18]. Therefore, motor activity could be particularly pronounced in the youngest boys, what teachers might mistake for hyperactive behaviour. Moreover, lower social-emotional skills of boys compared to girls [19] could lead to further problematic classroom behaviour. All this could result in teachers misinterpreting the behaviour of the youngest boys within a school class as indicative of ADHD more than that of the youngest girls. The fact that overdiagnosis of ADHD actually occurs in clinical practice has been already demonstrated for boys [20]. Similarly, under the stress-related hypothesis, one would expect more young boys within a grade to develop ADHD than young girls: Boys may experience more stress in school due to their lower school readiness levels compared to girls [16] and concerning genetic and environmental risk factors, the threshold for the induction of ADHD may be lower in boys compared to girls [21]. Taking these observations together, effect modification by gender could be an important aspect and should be thoroughly investigated.

To date, neither of the two hypotheses has been confirmed by available studies. Most studies used cross-sectional designs, only teacher reports, or administrative data, which are not helpful to clarify the nature of the association between ASE and ADHD diagnoses. To gain more evidence in this field, we analysed longitudinal data from a cohort study that examined the relationships between the child’s health and developmental status at school entry and long-term health and educational outcomes during primary school [22]. In the present report, we investigated the associations between ASE and parent-reported symptoms of attention-deficit/hyperactivity (ADH) prior to school entry and during first grade as well as teacher-reported symptoms of ADH at the end of first grade. According to the two possible explanations for the association, we created the following scenarios: If the relative age effect is due to oversupply, the younger and older children within a grade would already differ considerably in their ADH symptoms before and shortly after school entry. In contrast, if the effect is rather based on stress-related factors, the association between ASE and ADH symptoms should not be observed before and shortly after school entry, but would evolve over time.

## Methods

### The ikidS research project

ikidS (**i**ch **k**omme **i**n **d**ie **S**chule [German]: I am starting school) is an ongoing prospective population-based study with a closed cohort design [23]. The study investigated school beginners at 79 primary schools in the city of Mainz (i.e., the capital of the German Federal State of Rhineland-Palatinate) and the surrounding rural district of Mainz-Bingen. All parents of children in this region who had their mandatory preschool health examination (PHE) between 1st September 2014 and 31st August 2015 (*N* = 3683; population) were approached about study participation by public youth health physicians at the PHE and written informed parental consent was obtained. The research protocol was approved by the local ethics committee, the regional school authority, and the state representative for data protection. The sampling strategy, design aspects, response rates, and results on representativeness of the cohort have been described elsewhere [22].

### Exclusion criteria and analysis sample

Parents of 2003 children agreed to participate in ikidS (study sample, 54% of the population). By the end of first grade, the cohort comprised 1834 participants (due to withdrawal of consent, deferral from school entry, or migration). An additional 136 children were removed from the present analysis because they had not complied with the federal cut-off dates for school entry (i.e., 6th birthday between September 1st 2014 and August 31st 2015). This exclusion of children who have been deferred the year before (38 boys, 17 girls) or were too young for school entry (31 boys, 50 girls) according to legal regulations should prevent bias in the results. Another 65 children were excluded because neither the parents nor the teacher had responded to the questionnaires during first grade. This resulted in a final analysis sample of 1633 children (81.5% of the study sample; 44.3% of the population).

### Data collection and instruments

Data were collected at four-time points: at the PHE during the last preschool year (T0), 6 weeks before school entry (T1), 3 months after school entry (T2), and at the end of first grade (T3). At time points T1–T3, study-specific parental questionnaires were applied to investigate the child`s general and mental health as well as the child’s use of health services. At T3, class teachers completed a questionnaire about each child’s school-related behaviour. The response rate to the parental questionnaires ranged from 72.6% at T1 to 67.1% at T3 and was 82.9% for the teacher questionnaire (T3).

ADH symptoms were assessed at time points T1–T3 by parents and at T3 by class teachers using German versions of the Strengths and Difficulties Questionnaire (SDQ) for parents and teachers [24–26]. The SDQ is a widely used, validated five-scale screening instrument capturing behavioural problems and strengths of children and adolescents. ADH symptoms are covered by the hyperactivity/inattention subscale comprising 5 items which are rated on a 3-point scale (not true (0), somewhat true (1), certainly true (2)). The hyperactivity/inattention subscale score results from adding up the item scores and ranges from 0 to 10 points. Hence, a higher score indicates more ADH symptoms. The SDQ has already been applied successfully in several studies on the relative age effect [6, 12, 27]. As shown by the similar construct validity, both parents and teachers rate the same construct [28].

In addition to symptoms, the following clinical ADHD indicators were available from study-specific parental questionnaires: at time point T2, parents reported whether their child had a doctor’s diagnosis of ADHD or indications of a concentration disorder or hyperactivity. At T3, parents reported whether their child had a doctor’s diagnosis of ADHD or needed or used ADHD-related diagnostic procedures, whether their child had a prescription for ADHD medication or received such medication, and whether their child had behavioural problems.

We also used information from the parental questionnaires on the following potential risk factors for ADHD: gender, socio-economic status, migrant background, and family structure (nuclear family/single-parent family/foster parents/children’s home) [29]. This socio-demographic information was obtained with items and instruments retrieved from the German Health Interview and Examination Survey for Children and Adolescents [30].

### Statistical analysis

Demographic and clinical characteristics were described by appropriate statistical measures (e.g., numbers and frequencies for categorical variables and mean and standard deviation for continuous variables). All descriptive statistics were presented for complete cases. Representativeness was assessed by comparing the distribution of important demographic factors between the population and analysis sample.

ASE (in years) was defined as the difference between the date of the first day at school and the date of birth. For the primary analysis, a linear mixed model analysis for repeated measures was conducted with ASE as the independent variable and three reports of hyperactivity/inattention subscale as dependent variable: ratings from parents at T2 and T3 and from teachers at T3 were analysed as correlated outcomes (*N* = 1236, 1127, and 1393 of 1633, respectively). A separate linear regression analysis was carried out with baseline ratings of the hyperactivity/inattention subscale at T1 (prior to school entry) as the dependent variable. In both cases, effect estimates (non-standardized B- and standardized beta-coefficients) and their standard errors (SE) were calculated using different hierarchic models and adjustment sets: Model 1 included only gender. Model 2 included the adjustment set of model 1 plus socio-economic status assessed by Winkler’s index (range 0–21, higher scores reflect higher socio-economic status [31]), migrant background [32, 33], and family structure (nuclear family vs. other). Two other adjustment sets were only used in the linear mixed model analysis for repeated measures: Model 3 included the adjustment set of model 2 plus hyperactivity/inattention subscale at T1. Model 4 included the adjustment set of model 3 plus hyperactivity/inattention subscale at T2. In the case of model 4, the linear mixed model analysis for repeated measures was conducted only with ratings from parents and teachers at T3 as correlated outcomes. Missing data for the hyperactivity/inattention subscale and all used covariables were multiply imputed using the Monte Carlo Markov chain method (SAS procedure MI) assuming a multivariate normal distribution for outcome scores and covariables (10 imputations). The primary analysis was considered to be confirmatory; therefore, the level for type-1-error was set at 0.05.

Secondary analyses were performed to investigate the association between ASE and two relevant ADHD indicators: first, the hyperactivity/inattention subscale was dichotomised as either “no suspected ADHD” (score ≤ 5) or “suspected ADHD” (score > 5, borderline and abnormal range) by applying German population-based reference values [26]. Second, (parent reported) information on the clinical ADHD indicators, such as ADHD diagnosis, medication, or related diagnostic procedures, was combined into “no clinical indication of ADHD” and “clinical indication of ADHD”. The associations between ASE and suspected ADHD (based on the hyperactivity/inattention subscale) were investigated using marginal logistic regression analysis with generalised estimation equations by again combining assessments from parents (at T2, T3) and teachers (at T3) in one analysis (n as in the primary analysis). Odds ratios (OR) and their 95% confidence intervals (95% CI) were adjusted by the same adjustment sets (models 1–4) as in the primary analysis. The marginal logistic regression analysis with generalised estimation equations which used the clinical indication of ADHD as the dependent variable combined reports from parents at T2 and T3 in one analysis and was fitted only for the adjustment set of model 3. Missing values in outcomes and all used covariables were multiply imputed by the Monte Carlo Markov chain method. Full conditionals were specified as logistic models for all dichotomised SDQ hyperactivity/inattention subscale scores, using 100 imputations. The secondary analyses were considered to be exploratory; p-values were calculated only for descriptive purposes.

Due to obvious gender differences in the descriptive analysis concerning the relationship between ASE and the frequency of suspected ADHD, we additionally conducted a post hoc analysis. Here, a marginal logistic model for correlated outcomes was performed with (1) ASE as the independent variable, (2) the three dichotomised SDQ hyperactivity/inattention subscales (from parents at T2 and T3 and from teachers at T3) as dependent variables, and (3) an interaction term between ASE and gender. Odds ratios and their 95% confidence intervals were adjusted by the same variable sets as in models 1–4 of the primary analysis. All statistical analyses were carried out using SAS version 9.4.

## Results

Based on the PHE data, which were available for the entire population, Table 1 shows that the analysis sample was largely representative of the underlying population of first graders within the study region, apart from the migrant background (underrepresented) and maternal education (mothers with A-level exams overrepresented). The youngest quarter of the analysis sample showed some special features: it contained fewer boys and more children from nuclear families compared to the total analysis sample. However, when looking at the distribution of boys and girls across all ASE quarters, we found that boys were quite evenly distributed across quarters (youngest quarter 1: 24.4% of all boys; quarter 2: 25.2%; quarter 3: 24.9%; oldest quarter 4: 25.5%), while there was an imbalance across ASE quarters among girls. A slightly higher proportion of girls belonged to the youngest quarter, whereas a considerably lower proportion belonged to the oldest quarter (youngest quarter 1: 28.4% of all girls; quarter 2: 26.2%; quarter 3: 26.7%; oldest quarter 4: 18.7%).

**Table 1:** Characteristics of all school beginners within the study region (population), children included in the analysis sample, and children of the analysis sample belonging to the quarter with the youngest age at school entry^a^

Characteristics	Population *N* = 3683	Analysis sample *N* = 1633	Youngest quarter of analysis sample *N* = 430
Child			
Male, *n* (%)	1909 (51.9)	843 (51.6)	206 (47.9)
Missing, *n*	7	0	0
Migrant background, *n* (%)	822 (25.5)	325 (20.9)	91 (22.0)
Missing, *n*	464	80	17
Age at PHE, *M* (SD)	5.9 (0.4)	5.9 (0.3)	5.5 (0.2)
Missing, *n*	1	0	0
Family			
Family structure: nuclear family, *n* (%)	2855 (86.0)	1385 (86.9)	380 (90.0)
Missing, *n*	363	40	8
Multiples, *n* (%)	105 (2.9)	53 (3.3)	13 (3.0)
Missing, *n*	36	15	3
Abitur/Fachhochschulreife (A-level exams) Mother, *n* (%)	1825 (60.4)	941 (63.5)	255 (63.6)
Missing, *n*	662	150	29
Abitur/Fachhochschulreife (A-level exams) Father, *n* (%)	1768 (61.0)	874 (61.1)	236 (61.5)
Missing, *n*	784	203	46
Smoking in household			
Never, *n* (%)	3033 (91.9)	1455 (91.7)	374 (90.6)
Seldom, *n* (%)	191 (5.8)	96 (6.0)	28 (6.8)
Often, *n* (%)	77 (2.3)	36 (2.3)	11 (2.7)
Missing, *n*	382	46	17
Breastfeeding			
Not at all, *n* (%)	573 (17.5)	264 (16.8)	62 (15.1)
Up to 6 months, *n* (%)	1312 (40.2)	630 (40.2)	181 (44.1)
More than 6 months, *n* (%)	1382 (42.3)	674 (43.0)	167 (40.7)
Missing, *n*	416	65	20

*PHE* Preschool health examination, *M* Mean, *SD* Standard deviation

Percentages relate to non-missing values

^a^Age at school entry 6.0–6.25 years

The results of the primary analysis are presented in Table 2. There were no significant associations between ASE and the hyperactivity/inattention subscale prior to school entry (T1) and 3 months after school entry (T2). In contrast, a significant negative association was found at the end of the first grade (T3): depending on the adjustment set and observer, a one-year increase in ASE was associated with a decrease in the hyperactivity/inattention subscale of up to 0.96 score units (parent reports) or 1.34 score units (teacher reports).

**Table 2:** Association between age at school entry (independent variable) and the SDQ hyperactivity/inattention subscale (dependent variable) as assessed by linear mixed model analysis

	Baseline prior to school entry			Three months after school entry			End of first grade					
	Parent reports^*f*^			Parent reports^g^			Parent reports^g^			Teacher reports^g^		
	*B *^*SE*^	Beta^a^	*p*	*B *^*SE*^	Beta^a^	*p*	*B *^*SE*^	Beta^a^	*p*	*B *^*SE*^	Beta^a^	*p*
Model 1^b^	− 0.20^0.21^	− 0.03	0.34	− 0.19^0.20^	− 0.03	0.34	− 0.80^0.21^	− 0.10	< 0.001	− 1.13^0.25^	− 0.11	< 0.0001
Model 2^c^	− 0.37^0.21^	− 0.05	0.06	− 0.35^0.20^	− 0.05	0.09	− 0.96^0.21^	− 0.12	< 0.0001	− 1.34^0.25^	− 0.14	< 0.0001
Model 3^d^				− 0.10^0.16^	− 0.01	0.53	− 0.70^0.16^	− 0.09	< 0.0001	− 1.20^0.25^	− 0.12	< 0.0001
Model 4^e^							− 0.65^0.15^	− 0.08	< 0.0001	− 1.15^0.24^	− 0.12	< 0.0001

*B* Non-standardized regression coefficient, *SE* Standard error

^a^Standardized regression coefficient

^b^Adjusted for gender only

^c^Adjusted for gender, socio-economic status, migrant background, and family structure

^d^Adjusted for gender, socio-economic status, migrant background, family structure, and hyperactivity/inattention subscale at baseline

^e^Adjusted for gender, socio-economic status, migrant background, family structure, and hyperactivity/inattention subscale at baseline and 3 months after school entry

^b,c,d,e^Based on 1236 parent reports (3 months after school entry), 1127 parent reports (end of 1st grade), and 1393 teacher reports. Missing values in the four SDQ scores and all used covariables were multiply imputed by MCMC method assuming a multivariate normal distribution for outcome scores and covariables. 10 imputations

^f^A separate linear regression analysis was carried out with parent reports at T1 (baseline) as dependent variable and was fitted for adjustment set 1 and 2

^g^A linear mixed model analysis for repeated measures with parent reports at T2 and T3 and teacher reports at T3 (assessment) as correlated outcomes was fitted for each adjustment set, containing assessment and all interactions between assessment and all other variables as fixed effects

**Fig. 1:**
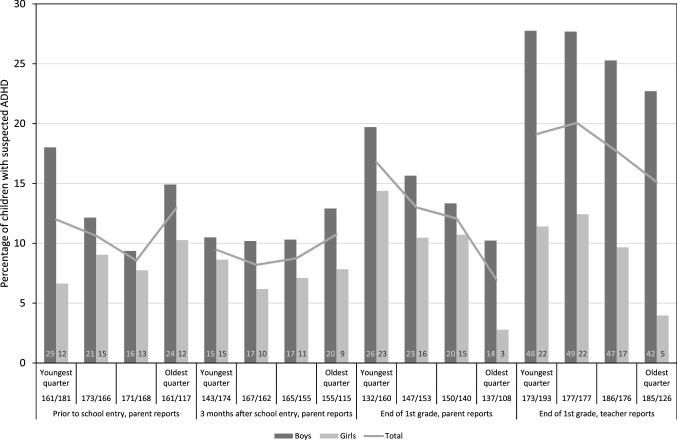
Percentage of children with SDQ hyperactivity/inattention subscale scores > 5 (“suspected ADHD”), presented separately for gender, time of observation, source of information (only at the end of first grade), and age at school entry, which is divided into quarters. Numbers in each bar indicate the respective number of children with suspected ADHD; numbers in the *x*-axis labelling indicate the corresponding sample sizes (*n* boys/*n* girls)

The proportion of children with suspected ADHD based on the hyperactivity/inattention subscale (scores higher than 5) at the different assessment points ranged from 11.0 to 14.7% among boys and from 7.4 to 10.2% among girls (parent reports). These proportions are in line with the recently published age- and gender-specific norm values for the German SDQ hyperactivity/inattention subscale [34], which is further support for the assumption that our analysis sample is representative. Further frequencies of suspected ADHD are given in the Online Resource (Table S1). Figure 1 depicts the proportion of children with suspected ADHD for each assessment point separately for ASE quarters. On a descriptive level, results show that the proportion of children with suspected ADHD increased in the course of the first school year and that the proportion of children with suspected ADHD was higher among boys than among girls at all assessment points and for all age segments. At T3, ADHD was suspected most frequently in the youngest quarter of the children (parent-reported ADH symptoms) or in the younger half (teacher-reported ADH symptoms). Differences in the proportion of suspected ADHD between younger and older children were more pronounced in parent reports compared to teacher reports. It is also noticeable in Fig. 1 that both in parent and teacher reports at T3, girls showed larger differences in the proportion of suspected ADHD between the youngest and oldest quarter than boys, thus, suggesting some interaction with gender.

The results of the secondary analysis confirmed the association between ASE and suspected ADHD based on the hyperactivity/inattention subscale. As presented in Table 3, a one-year increase in ASE significantly reduced the odds of suspected ADHD at time point T3 by up to 75% (parent reports) or 54% (teacher reports). In analogy to the primary analysis, this association was not yet present at T2.

**Table 3:** Associations between age at school entry (independent variable) and the dichotomised SDQ hyperactivity/inattention subscale (dependent variable; suspected ADHD with scores > 5) as assessed by logistic regression analysis^a^

	Three months after school entry		End of first grade			
	Parent reports		Parent reports		Teacher reports	
	Odds ratio (95% CI)	*p*	Odds ratio (95% CI)	*p*	Odds ratio (95% CI)	*p*
Model 1^b^	1.13 (0.57, 2.23)	0.72	0.30 (0.16, 0.57)	< 0.0002	0.56 (0.34, 0.91)	0.018
Model 2^c^	0.93 (0.46, 1.88)	0.84	0.27 (0.14, 0.51)	< 0.0001	0.46 (0.28, 0.77)	0.003
Model 3^d^	1.06 (0.49, 2.29)	0.88	0.25 (0.12, 0.50)	< 0.0001	0.47 (0.28, 0.80)	0.005
Model 4^e^			0.21 (0.10, 0.45)	< 0.0001	0.46 (0.27, 0.79)	0.005

*CI* confidence interval

^a^A marginal logistic model for correlated outcomes (parent reports at T2, T3, teacher reports at T3; assessment) was fitted for each adjustment set, containing assessment and all interactions between assessment and all other variables as fixed effects

^b^Adjusted for gender only

^c^Adjusted for gender, socio-economic status, migrant background, and family structure

^d^Adjusted for gender, socio-economic status, migrant background, family structure, and hyperactivity/inattention subscale at baseline

^e^Adjusted for gender, socio-economic status, migrant background, family structure, and hyperactivity/inattention subscale at baseline and 3 months after school entry

^b,c,d,e^Based on 1236 parent reports (3 months after school entry), 1127 parent reports (end of 1st grade), and 1393 teacher reports. Missing values in outcomes and adjustment set (c) multiply imputed by MCMC method. Full conditionals specified as logistic models for all dichotomized SDQ hyperactivity/inattention subscale scores, using 100 imputations

The proportion of children with a clinical ADHD indication was somewhat smaller than the proportion of children with suspected ADHD based on the hyperactivity/inattention subscale, especially according to parent-reported indicators at T3 (5.3% for boys, 2.5% for girls). Also for the clinical indicators, the proportion of children with ADHD indication was higher among boys than among girls. Further results are listed in the Online Resource (Table S1). Regarding the logistic regression analysis using this clinical ADHD indication as the dependent variable, there was no significant association with ASE, neither at T2 (OR = 0.83; 95% CI 0.37, 1.85; *p* = 0.64) nor at T3 (OR = 1.20; 95% CI 0.45, 3.19; *p* = 0.72; results of the fully adjusted model).

Because of the observed modifying effect of gender that was apparent on the descriptive level, we conducted a post hoc analysis to examine whether the changes in the ASE effect from school entry to the end of the first grade were mainly driven by girls. The results, which are given in Table 4, are consistent across all models: again, there was no significant association between ASE and suspected ADHD based on the hyperactivity/inattention subscale at T2, neither for girls nor for boys. Parent reports at T3 showed a significant association with ASE that was descriptively stronger for girls than for boys, but the interaction with gender missed statistical significance. Suspected ADHD based on teacher reports at T3 showed a significant association with ASE only for girls, and for models 2, 3, and 4, the interaction with gender was significant. Thus, in this case, the ASE effect seemed to differ between girls and boys; a one-year increase in ASE reduced the odds of suspected ADHD by 81% for girls compared to 30% for boys (model 3). When analysing gender-specific effects of ASE on the continuous (non-dichotomous) SDQ hyperactivity/inattention subscale, the effect appeared generally more pronounced in girls than in boys, but the interaction with gender did not reach significance in any model (results not shown).

**Table 4:** Gender-specific associations between age at school entry (independent variable) and the dichotomised SDQ hyperactivity/inattention subscale (dependent variable; suspected ADHD with scores > 5) as assessed by logistic regression analysis^a^

	Three months after school entry		End of first grade			
	Parent reports		Parent reports		Teacher reports	
	Odds ratio (95% CI)	*p* (Interaction)^f^	Odds ratio (95% CI)	*p* (Interaction)^f^	Odds Ratio (95% CI)	*p* (Interaction)^f^
Model 1^b^						
Boys	1.15 (0.47, 2.82)	0.98	0.38 (0.17, 0.84)*	0.34	0.72 (0.40, 1.28)	0.092
Girls	1.13 (0.38, 3.34)		0.21 (0.08, 0.56)**		0.30 (0.13, 0.70)**	
Model 2^c^						
Boys	1.02 (0.41, 2.56)	0.78	0.35 (0.16, 0.78)*	0.27	0.64 (0.35, 1.19)	0.041
Girls	0.82 (0.27, 2.54)		0.17 (0.06, 0.47)***		0.21 (0.09, 0.52)***	
Model 3^d^						
Boys	1.42 (0.51, 3.96)	0.39	0.39 (0.17, 0.93)*	0.092	0.70 (0.37, 1.33)	0.020
Girls	0.70 (0.20, 2.36)		0.12 (0.04, 0.37)***		0.19 (0.07, 0.47)***	
Model 4^e^						
Boys			0.32 (0.13, 0.82)*	0.15	0.67 (0.35, 1.28)	0.027
Girls			0.11 (0.03, 0.36)***		0.18 (0.07, 0.48)***	

*CI*  confidence interval

*p < 0.05

**p < 0.01

***p < 0.001

^a^A marginal logistic model for correlated outcomes (parent reports at T2, T3, teacher reports at T3; assessment) was fitted for each adjustment set, containing assessment and all interactions between assessment and all other variables as fixed effects

^b^Unadjusted

^c^Adjusted for socio-economic status, migrant background, and family structure

^d^Adjusted for socio-economic status, migrant background, family structure, and hyperactivity/inattention subscale at baseline

^e^Adjusted for gender, socio-economic status, migrant background, family structure, and hyperactivity/inattention subscale at baseline and 3 months after school entry

^b,c,d,e^Based on 1236 parent reports (3 months after school entry), 1127 parent reports (end of 1st grade), and 1393 teacher reports. Missing values in outcomes and adjustment set (c) multiply imputed by MCMC method. Full conditionals specified as logistic models for all dichotomized SDQ hyperactivity/inattention subscale scores, using 100 imputations

^f^Chi-square test for interaction between gender and age at school entry

## Discussion

The results of the present study show a negative association between ASE and ADH symptoms at the end of first grade, but not before. The younger children within a grade had increased odds of borderline or abnormal scores on the hyperactivity/inattention subscale of the SDQ. This association persisted regardless of the source of information and remained after adjusting for potential confounders and ADH symptoms at baseline (prior to school entry) as well as 3 months after school entry. In contrast, ASE was not associated with parent-reported ADH symptoms prior to school entry and 3 months after school entry. At the end of first grade, the association between young ASE and more ADH symptoms in the borderline or abnormal range was consistently more pronounced for girls than for boys. The observed effect modification of ASE by gender was significant only in the case of teacher-reported ADH symptoms. Regarding the clinical ADHD indications during first grade, such as the use of ADHD-related diagnostic procedures, a doctor’s diagnosis of ADHD, or related medication, we found no association with ASE.

In the light of the pre-specified two hypotheses (oversupply vs. stress-related) and the expectations concerning the presence of associations between ASE and ADHD-related outcomes prior to and after school entry, our results provide some clues supporting the stress-related hypothesis as an explanation for the relative age effect on ADHD-related outcomes. The finding that more ADH symptoms were reported for the younger children than for the older children at the end of first grade, but not yet around school entry, suggests that the increase in symptoms could be a consequence of school entry in a group of susceptible young first graders. Moreover, the fact that the relative age effect occurred not only in teacher reports but also in parent reports of ADH symptoms, makes it less likely that it is based solely on teachers` misinterpretation of immature behaviours. Thus, our results better fit the stress-related hypothesis than the oversupply hypothesis. For the latter, we would expect that the differences in ADH symptoms between younger and older children within a grade should be present and already perceptible around school entry.

However, these results of an observational study could not be interpreted as proof of one of the hypotheses. Since it is not possible to conduct a randomized-controlled trial with a control group of the same age that is not enrolled in school, no clear causal inference can be drawn, yet. There are several other conceivable reasons for the absence of an association between ASE and ADH symptoms at the two earlier time points around school entry. One of them could be, for example, that the measure is not sufficiently sensitive for young children, another, that the development of the prefrontal brain regions does not proceed continuously and, therefore, younger and older children do not differ equally in their inattentive, hyperactive, and impulsive behaviour at all points in time. Hence, the present pattern of results cannot definitely decide between the two hypotheses.

Notwithstanding these concerns, our results are largely consistent with previous studies on the relationship between ASE and ADH symptoms [4–7], but most of these studies did not address the nature of the association. Usually, studies only compared the relative risk of an ADHD diagnosis or medication between younger and older children within a grade, e.g., [35–37], or between the youngest of a grade (born in the last month before the school entry cut-off date) and the oldest of the next higher grade (born immediately in the month after the cut-off date; e.g., [3, 7, 38]). In a study by Elder [7], the ASE effect was stronger based on teacher reports of ADHD symptoms compared to the ASE effect based on parent reports. The author concluded that the teachers’ perceptions of a child’s behaviour are parts of the mechanism underlying the association between ASE and ADHD diagnoses, which would—at least partially—support the oversupply hypothesis.

However, findings are somewhat mixed with regard to differences between teacher and parent ratings of ADH symptoms. Besides some studies reporting weaker ASE effects for parent ratings compared to teacher ratings [6, 7, 12] or even no effect at all [38], Muehlenweg et al. [4] showed an effect of ASE on parent-reported hyperactivity symptoms while controlling for the level of symptoms prior to school entry. This is similar to our study, where—based on the point estimates—the ASE effect was in part even stronger for parent ratings than for teacher ratings. The repeated finding of an association between ASE and parent-reported ADH symptoms seems to challenge the view that the relative age effect is only caused by teachers’ biased assessments resulting in more ADHD-related health care use by counselling parents in that way. However, it cannot be ruled out that parent ratings given at the end of first grade are already affected by the teachers’ perspective and the parent-teacher communication during first grade. As a result, parents of younger children may report more symptoms than they would have done without this communication. The validity of ADH symptom ratings from teachers and parents during first grade and the effect of parent-teacher communication on the initiation of ADHD-related health care use should be definitively clarified in future studies. On the other hand, the finding of a stronger relative age effect on teacher-reported ADH symptoms does not necessarily support the relevance of the oversupply hypothesis. It is also conceivable that ADH symptoms may be more pronounced in the school context compared to the home environment or that teachers assess a child’s extrinsic behaviour more accurately compared to parents, whose ratings may be affected by a social desirability bias [7].

Our results tentatively suggest a gender-specific ASE effect, which is in line with other studies also showing a more pronounced relative age effect in girls compared to boys [36, 37, 39], although in these studies—as in ours—the proportion of actual or suspected cases of ADHD was higher among boys than among girls. On the basis of both the oversupply and the stress-related hypothesis, we would have expected the ASE effect to be stronger in boys because of the greater immaturity of boys compared to girls [15]. However, our results did not confirm this expectation. The reason for the more pronounced ASE effect observed in girls in our study remained unclear. One possible explanation could be that boys lag so far behind girls in maturity that even the older boys within a grade are developmentally less mature than the younger girls, and therefore the maturity difference between the younger and older boys does not have the same effect as for the girls. Another explanation in the context of the stress-related hypothesis could be based on the assumption that girls take school more seriously from the beginning and put themselves under more pressure than boys. As a consequence, especially the younger and developmentally more immature girls may experience more stress at school, which in turn may trigger or amplify ADHD symptoms. Indeed, there are studies on school-related stress among adolescent pupils, in which girls reported to feel a higher pressure to perform at school than boys did and also reported more psychosomatic symptoms than boys [40, 41]. Already at primary school age, girls reported a higher vulnerability to stress and more stress symptoms than boys [42]. We recommend further elucidate the reason for the stronger relative age effect among girls. For this goal, qualitative studies including semi-structured interviews may be helpful.

In contrast to many other studies reporting a difference in the risk of ADHD diagnosis and medication between the youngest and oldest children within a school class [37, 38, 43, 44], a corresponding effect was not observed in our study involving first graders. This is in line with another study where the association between relative ASE and rates of ADHD diagnoses and medications was also not evident in first grade but was detectable from grade three onwards [3]. This could be explained by an expected delay between the occurrence and recognition of early symptoms and the use of related health care services. In a Norwegian study, the relative age effect also did not appear before grade three [36]. In contrast, a Danish study found no relative age effect at all on ADHD medication, which the authors attribute to the high proportion of young children with deferred school entry and the low prescription rates for ADHD medication in Denmark [45].

Taken these and our findings into consideration, the relative age effect on ADHD-related outcomes likely depends on amplifying and mitigating country-specific contextual factors like school entry policy, early school-related demands, the role of teachers in perceiving ADHD symptoms and counselling parents, as well as ADHD-related health care properties. It is crucial to determine the precise causal mechanism underlying this phenomenon to develop appropriate strategies tackling the undesirable consequences: in case of oversupply, teachers and clinicians should be sensitised to the relative age of a child within his or her class and the associated danger of misjudging immature but age-appropriate behaviour [2]. Clinicians should strictly adhere to accepted diagnostic criteria and use structured diagnostic interviews to avoid overdiagnosis [20]. Teachers should adjust their educational activities and academic expectations to the developmental status of the child [12, 27]. In the case of young ASE actually causing or promoting ADHD-related outcomes, more attention should be paid to address ASE as an important and modifiable risk factor for ADHD. The exact biological mechanism should then be clarified and on this basis, effective prevention strategies should be developed. Consequently, children at risk for developing ADHD or related symptoms later in school should be identified prior to school entry. This could be done by screening for ADHD risk factors and specific neuropsychological deficits predictive of later ADHD [46, 47]. Such risk assessments could be integrated into existing PHE concepts to initiate further measures, such as delaying school entry or providing individual support for children at risk. In conclusion, we are far from understanding this phenomenon, and it is of high scientific interest and practical importance to solve the mystery of the relative age effect.

### Strengths and limitations

The major strengths and limitations of the ikidS cohort study have been extensively discussed elsewhere [22]. The present study stands out due to the large population-based and representative sample, the prospective, longitudinal design with three data collection points, and the adjustment for pre-existing ADH symptoms and other potential confounders in the analysis. However, the following limitations should be considered: ADH symptoms were assessed by the hyperactivity/inattention subscale of the SDQ, which cannot be interpreted as proof of an ADHD diagnosis. This subscale measures hyperactive, inattentive, and impulsive behaviour that may also occur for reasons other than ADHD (e.g., sleep problems with daytime sleepiness, trauma, bullying, difficult family situation, learning disorders, or other mental disorders, such as depression, anxiety disorders, psychoses, or developmental disorders). Therefore, the increase in ADH symptoms in young children does not necessarily correspond to an increase in ADHD, but could also reflect an increase in other problems. Notwithstanding this concern, a validation study demonstrated that the hyperactivity/inattention subscale of the German version of the SDQ was able to discriminate between patients with and without a diagnosis of ADHD and that it is useful for screening purpose [48]. To capture clinical ADHD indicators, validated instruments or objective data (e.g., administrative data) were not available. The use of ADHD indicators based on items of study-specific parental questionnaires revealed only a small numbers of cases, which led to an underpowered analysis of the clinical ADHD indications.

Another limitation concerns the obvious selection of children for school entry due to the flexible school enrolment practice in Rhineland-Palatinate. Here, the PHE serves to assess the school readiness of all preschool children and, among other things, to recommend early or deferred school entry. This usually results in fewer young girls being deferred for 1 year compared to young boys and more of the oldest girls being allowed to start school 1 year earlier compared to the oldest boys. This had effects on our analysis sample showing (1) only a negligible selection among boys, but (2) a slightly stronger selection among girls in the youngest quarter, and (3) a pronounced selection among girls in the oldest quarter. Theoretically, this could create a spurious association between ASE and ADH symptoms if—and only if—older girls with ADH symptoms are generally selected for early school enrolment the year before. In this case, these symptomatic girls would not be part of the class 1 year later and this would lead to the impression that younger girls have more ADH symptoms compared to older girls within the class. In fact, the opposite is more likely: older and therefore developmentally more mature girls with fewer ADH symptoms are expected to be selected for early school enrolment, leading to a larger number of older girls with ADH symptoms within the class. Despite this potential attenuating influence of the school enrolment practice on the ASE effect, we were able to confirm the presence of such an effect in girls. Hence, we believe that the observed ASE effect was not artificially created by selection bias.

## Conclusions

The youngest children within first grade show more parent- and teacher-reported ADH symptoms compared to their older classmates. This relative age effect is not seen around school entry, which may provide some first indications that, among other explanations, stress-related factors may account for at least some part of the association. Parents, teachers, and clinicians should be sensitised to this relative age effect to avoid oversupply, misdiagnosis and, perhaps, unnecessary ADHD-related medication. The true mechanism of the association should be discovered to enable effective preventive interventions, which should lead to lower rates of ADHD diagnoses and medications in relatively young primary school children.

## Supplementary Information

Below is the link to the electronic supplementary material.

Supplementary Table S1 Frequency of ADHD indications or suspected ADHD based on the different ADHD variables in the analysis sample (DOCX 14 KB)
