# Supplementary material for: Age at school entry and reported symptoms of attention-deficit/hyperactivity in first graders: results of the prospective cohort study ikidS
Source: Eur Child Adolesc Psychiatry. 2021 Jun 5;31(11):1753–64. doi: 10.1007/s00787-021-01813-7 (PMC9666310; doi:10.1007/s00787-021-01813-7)
Supplement: Supplementary file 1 — Supplementary Table S1 Frequency of ADHD indications or suspected ADHD based on the different ADHD variables in the analysis sample (DOCX 14 KB) [file 787_2021_1813_MOESM1_ESM.docx]

Table S1

*Frequency of ADHD indications or suspected ADHD based on the different ADHD variables in the analysis sample*

|  | Boys (N = 843) | | | | Girls (N = 790) | |
| --- | --- | --- | --- | --- | --- | --- |
|  | | N | Suspected ADHD /  ADHD indication  n (%) | N | | Suspected ADHD /  ADHD indication  n (%) |
| SDQ-H/I subscale^a^, parent reports at T1 | | 666 | 90 (13.51) | 632 | | 52 (8.23) |
| SDQ-H/I subscale^a^,  parent reports at T2 | | 630 | 69 (10.95) | 606 | | 45 (7.43) |
| SDQ-H/I subscale^a^,  parent reports at T3 | | 566 | 83 (14.66) | 561 | | 57 (10.16) |
| SDQ-H/I subscale^a^,  teacher reports at T3 | | 721 | 186 (25.80) | 672 | | 66 (9.82) |
| Clinical ADHD indicators,  parent reports at T2^b^ | | 644 | 66 (10.25) | 615 | | 36 (5.85) |
| Clinical ADHD indicators,  parent reports at T3^c^ | | 568 | 30 (5.28) | 568 | | 14 (2.46) |

SDQ-H/I subscale = SDQ hyperactivity/inattention subscale.

T1: baseline prior to school entry; T2: three months after school entry; T3: end of first grade.

^a^ ADHD was suspected with scores > 5.

^b^ Indication of ADHD was given if parents reported that their child had a doctor’s diagnosis of ADHD or a concentration disorder or hyperactivity.

^c^ Indication of ADHD was given if parents reported that their child had a doctor’s diagnosis of ADHD or that ADHD-related diagnostic procedures were used or that their child has received ADHD medication or that their child had social behaviour problems.
